# Draft Genome Sequences of Two Gammaproteobacterial Methanotrophs Isolated from Rice Ecosystems

**DOI:** 10.1128/genomeA.00526-17

**Published:** 2017-08-17

**Authors:** Katharina Frindte, Marina G. Kalyuzhnaya, Françoise Bringel, Peter F. Dunfield, Mike S. M. Jetten, Valentina N. Khmelenina, Martin G. Klotz, J. Colin Murrell, Huub J. M. Op den Camp, Yasuyoshi Sakai, Jeremy D. Semrau, Nicole Shapiro, Alan A. DiSpirito, Lisa Y. Stein, Mette M. Svenning, Yuri A. Trotsenko, Stéphane Vuilleumier, Tanja Woyke, Claudia Knief

**Affiliations:** a Institute of Crop Science and Resource Conservation—Molecular Biology of the Rhizosphere, University of Bonn, Bonn, Germany; b San Diego State University, San Diego, California, USA; c Department of Microbiology, Genomics and the Environment, Université de Strasbourg, UMR 7156 CNRS, Strasbourg, France; d Department of Biological Sciences, University of Calgary, Calgary, Alberta, Canada; e Department of Microbiology, Faculty of Science, Radboud University, Nijmegen, The Netherlands; f G.K. Skryabin Institute of Biochemistry and Physiology of Microorganisms, Russian Academy of Sciences, Pushchino, Russia; g Division of Mathematics and Natural Sciences, Queens College of the City University of New York, Flushing, New York, USA; h School of Environmental Sciences University of East Anglia, Norwich, United Kingdom; i Division of Applied Life Sciences, Graduate School of Agriculture, Kyoto University, Kitashirakawa-Oiwake, Kyoto, Japan; j Department of Civil and Environmental Engineering, University of Michigan, Ann Arbor, Michigan, USA; k DOE Joint Genome Institute, Walnut Creek, California, USA; l Roy J. Carver Department of Biochemistry, Biophysics and Molecular Biology, Iowa State University, Ames, Iowa, USA; m Department of Biological Sciences, University of Alberta, Edmonton, Alberta, Canada; n Department of Arctic and Marine Biology, UiT The Arctic University of Norway, Tromsø, Norway

## Abstract

The genomes of the aerobic methanotrophs “*Methyloterricola oryzae*” strain 73a^T^ and *Methylomagnum ishizawai* strain 175 were sequenced. Both strains were isolated from rice plants. *Methyloterricola oryzae* strain 73a^T^ represents the first isolate of rice paddy cluster I, and strain 175 is the second representative of the recently described genus *Methylomagnum*.

## GENOME ANNOUNCEMENT

Aerobic methanotrophic bacteria play a key role in controlling global climate by reducing the emission of the greenhouse gas methane in ecosystems such as paddy fields (1). Gammaproteobacterial methanotrophs are common inhabitants of rice fields (2, 3). We sequenced the genomes of two gammaproteobacterial isolates from rice plants (4). “*Methyloterricola oryzae*” strain 73a^T^ (=LMG 29185=VKM-B-2986) is currently the only cultivated representative of rice paddy cluster I (3, 5), and *Methylomagnum ishizawai* strain 175 (= LMG 28717=VKM-B-2989) is the second representative of the genus (6).

Genomic DNA was extracted from bacterial cultures using a phenol-chloroform method (7), and draft genome sequences were generated at the DOE Joint Genome Institute. The genome of strain 73a^T^ was sequenced using an Illumina HiSeq 2000, which generated 11,844,428 reads (1.79 Gb). The Pacific Biosciences RS was used for strain 175, and 230,505 filtered subreads (0.73 Gb) were generated. Sequence filtering, genome assembly, and gene annotation were performed as described earlier (8, 9). The final draft of strain 73a^T^ had 302.0× read coverage, contained 74 contigs in 73 scaffolds, was 4.9 Mb in size, and had an average GC content of 61.1%. The draft of strain 175 had 140.8× read coverage, contained 8 contigs in 8 scaffolds, was 5.5 Mb in size, and had an average GC content of 63.0%.

Both strains encode metabolic inventory typical for type I methanotrophs (10). They harbor genes encoding a particulate methane monooxygenase (*pmoCAB*) and a methane/ammonia monooxygenase-related protein (*pxmABC*) (11). Additionally, the genome of strain 175 encodes a soluble methane monooxygenase (*mmoXYBZDCGR*). Gene clusters for PQQ-dependent methanol dehydrogenases and PQQ biosynthesis were found in both strains (*mxaFJGIRSACKLD*, *xoxFJ*, *pqqABCDE*, and *pqqFG*). Formaldehyde oxidation is predicted to proceed via the tetrahydromethanopterin (H_4_MPT) pathway (presence of *fae*, *mtdB*, *mch*, *fhcABCD*, and H_4_MPT biosynthesis genes) (12). Additionally, both strains contained genes encoding the tetrahydrofolate-dependent pathway (presence of *mtdA*, *fchA*, *fhs*, and, additionally, a *folD* gene in strain 73a^T^). Formate can potentially be oxidized via one (strain 175) or two (strain 73a^T^) types of formate dehydrogenase.

Both strains may assimilate formaldehyde via the ribulose monophosphate pathway. The cleavage cascade can be realized via fructose-1,6-bisphosphate, and in strain 73a^T^ additionally via 2-keto-3-deoxy-6-phosphogluconate. Rearrangement of ribulose-5-phosphate can occur by transketolase and transaldolase reactions. A complete serine cycle is unlikely to be present. Both strains have the genes necessary for operational oxidative pentose phosphate and TCA cycle pathways, whereas a complete glycolysis cascade is encoded only in strain 73a^T^. RubisCO genes are present (*cbbL* and *cbbS* in strain 175; *cbbM* in strain 73a^T^), as well as genes for a complete Calvin-Benson-Bassham cycle.

For nitrogen acquisition, both strains possessed genes encoding ammonium (*amtB*), nitrate (*nasA*), and urea (*urtABCDE*) transporters, as well as urease genes (*ureABCDEFG*). Moreover, *nif* genes were present, suggesting the potential for dinitrogen fixation. The strains may form polyphosphate (*ppk*) and glycogen (*glgAB*, *glgC, glgP, glgX*, *malQ*, and *pgm*) as storage compounds. Strain 175, in addition, can potentially produce polyhydroxybutyrate (*phbAB* and *phbC*), a characteristic not yet known for type I methanotrophs (13).

### Accession number(s).

The genome sequences have been deposited in GenBank under the accession numbers JYNS00000000, for *Methyloterricola oryzae* strain 73a^T^, and FXAM00000000, for *Methylomagnum ishizawai* strain 175.
